# Bone and Bone Marrow Involvement in Sarcoidosis

**DOI:** 10.4274/tjh.2013.0255

**Published:** 2014-06-10

**Authors:** Gökhan Sargın, İrfan Yavaşoğlu, Gürhan Kadıköylü, Zahit Bolaman

**Affiliations:** 1 Adnan Menderes University Faculty of Medicine, Department of Internal Medicine, Aydın, Turkey; 2 Adnan Menderes University Faculty of Medicine, Department of Hematology, Aydın, Turkey

**Keywords:** Sarcoidosis, Bone, Bone marrow

## TO THE EDITOR

We report a 52-year-old male patient with bone marrow and skeletal system involvement of sarcoidosis. In sarcoidosis, while bone involvement occurs at a rate of 10%-15%, iliac bone and bone marrow involvement is less common [1,2].

**Figure 1:**
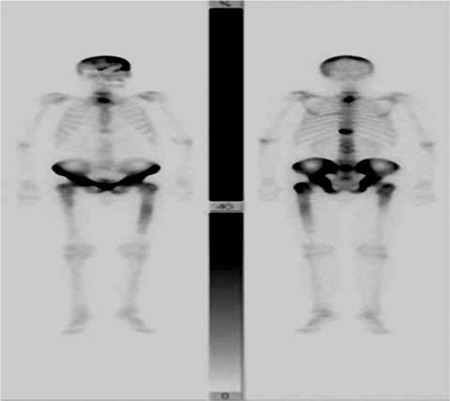
Dense and diffuse increased osteoblastic activity in the cranium; all pelvic and sacroiliac joints; the C7, T1-2, and T11 vertebrae of the vertebral column; and both proximal femurs on technetium-99m bone scintigraphy.

**Figure 2:**
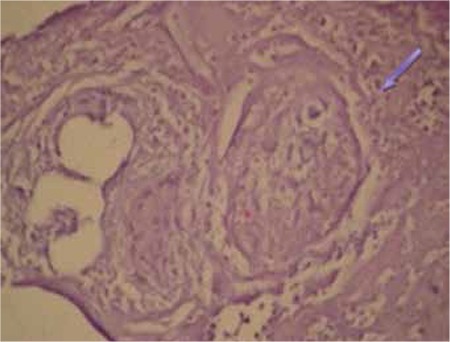
Noncaseating granulomatous inflammation in the bone marrow biopsy (hematoxylin & eosin, 100x).

A 52-year-old male patient was admitted to our clinic with increased lower back pain with activity for the last 2 months. The patient occasionally coughed up white sputum. He had a 20-year medical history of sarcoidosis, but had received no treatment for the last 5 years. There were bilateral crackles in the middle and lower zones of the lungs. The sacroiliac tension test was positive. Erythrocyte sedimentation rate was 42 mm/h, hemoglobin 14.5 g/dL, hematocrit 43.8%, leukocyte count 7100/mm3, platelet count 376,000/mm3, serum calcium 9.6 mg/dL, total prostate-specific antigen 2.7 ng/mL, total protein 6.8 g/dL, and albumin 3.3 g/dL; the serum alkaline phosphatase level was normal. The tuberculin skin test result was 8 mm and sputum smear examinations for acid-resistant bacilli were negative for 5 times. Polyclonal gammopathy was detected in protein electrophoresis. While in hip radiography increased cortical thickness was observed in the bone pelvis and femur, in cervical and cranial radiography, bone structures and soft tissues were normal. Posteroanterior chest radiography revealed diffuse reticular infiltration. Thoracic computed tomography was consistent with stage 4 sarcoidosis. Technetium-99m bone scintigraphy revealed dense and diffuse increased osteoblastic activity in the cranium; all pelvic and sacroiliac joints; C7, T1-2, and T11 vertebrae of the vertebral column; and both proximal femurs (Figure 1). There were no pathological or radiological findings upon magnetic resonance imaging of the lumbosacral region. These findings were found to be concordant with skeletal system involvement of sarcoidosis. Bone marrow biopsy was performed due to increased osteoblastic activity on technetium-99m bone scintigraphy and noncaseating granulomatous inflammation was detected (Figure 2). A distinct feature of sarcoidosis is the local accumulation of inflammatory cells. Based on bone marrow biopsy and imaging results of technetium-99m bone scintigraphy, the patient was diagnosed with vertebral, iliac bone, and bone marrow involvement of sarcoidosis, and treatment with 1 mg/kg/day methylprednisolone was started. Systemic corticosteroids remain the mainstay of treatment. However, clinicians should be careful with methotrexate due to its cytotoxic effects on bone marrow. We could not reevaluate the response to treatment with technetium bone scintigraphy. The patient is still under our follow-up without any symptoms. Informed consent was obtained.

In sarcoidosis, bone and bone marrow involvement should be kept in mind in patients with lower back pain.

## ACKNOWLEDGMENTS

For their contributions to the images, we thank Füruzan Döger (Adnan Menderes University Faculty of Medicine, Department of Pathology) and Yakup Yürekli (Adnan Menderes University Faculty of Medicine, Department of Nuclear Medicine).

## CONFLICT OF INTEREST STATEMENT

The authors of this paper have no conflicts of interest, including specific financial interests, relationships, and/ or affiliations relevant to the subject matter or materials included.
